# Use of Sildenafil Citrate for the Treatment of Erectile Dysfunction in Stallions: First Case Reports

**DOI:** 10.1111/rda.70318

**Published:** 2026-09-08

**Authors:** Thaís Mendes Sanches Cavalero, Frederico Ozanam Papa, Gabriel Augusto Monteiro, Patrícia de Mello Papa, Felipe Pires Hartwig, Camila de Paula Freitas‐Dell'Aqua, Marco Antônio Alvarenga, Marcela Souza e Freitas, Yame Fabres Robaina Sancler‐Silva

**Affiliations:** ^1^ Department of Veterinary Surgery and Animal Reproduction, School of Veterinary Medicine and Animal Science UNESP Botucatu São Paulo Brazil; ^2^ Department of Animal Science Federal University of Viçosa, UFV Viçosa Minas Gerais Brazil

**Keywords:** chemical ejaculation, penile erection, semen, stallion sexual behaviour

## Abstract

Pharmacological aids can be useful alternatives to enhance penile erection and/or improve ejaculatory function, thereby prolonging the reproductive life of stallions with ejaculatory disorders. This study aimed to evaluate the effects of sildenafil citrate in the treatment of erectile dysfunction and on semen quality in two aged stallions, a Quarter Horse (18 years) and a Mangalarga Marchador (25 years), presented to the Veterinary Hospital of FMVZ‐UNESP, Botucatu, SP, Brazil. The treatment consisted of oral administration of sildenafil citrate at 0.2 mg/kg. Thirty minutes after administration, the Quarter Horse stallion achieved full erection and ejaculated in all collection attempts (100%; 3/3), with semen parameters within normal ranges for the species. The Mangalarga Marchador showed a partial response to treatment, with successful ejaculation in only one of three attempts (33.3%) and exhibited oligozoospermia, possibly associated with previously identified age‐related testicular degeneration concurrent with the erectile dysfunction. To the best of the authors' knowledge, this is the first report on the use of sildenafil citrate for managing erectile dysfunction in stallions, demonstrating its therapeutic potential. However, the results suggest individual variability in response to treatment, indicating the need for additional controlled studies to establish more precise dose–response protocols.

## 1 Introduction

Various factors can influence erection, emission and ejaculation in stallions, and these are often associated with ejaculatory disorders or the inability of stallions to perform breeding. Difficulties in copulation are frequently related to low libido, erectile deficits, neurological problems, penile injuries or neoplasms and musculoskeletal issues. Although erection is not essential for ejaculation, stallions with erectile dysfunction often exhibit ejaculatory difficulties (McDonnell 2000).

Copulatory and ejaculatory disorders in stallions have been recognized for decades, and different diagnostic and therapeutic approaches have been proposed for their management (Klug et al. 1999). Pharmacological protocols involving imipramine, α‐adrenergic agonists and oxytocin have been used primarily to facilitate emission and ejaculation in stallions with ejaculatory dysfunction (McDonnell 2001; Cavalero et al. 2020; Garza et al. 2025). However, these therapies act predominantly on the neurophysiological mechanisms involved in emission and ejaculation (McDonnell 2001; Monteiro and Petelinkar 2025) and may therefore have limited efficacy when reproductive impairment is primarily related to the inability to achieve or maintain an adequate erection. In such cases, pharmacological approaches aimed at enhancing penile blood flow may represent a more specific therapeutic strategy to improve erectile function and enable successful copulation.

Sildenafil citrate (SC) is a widely used and effective on‐demand medication for men with erectile dysfunction. In addition to its proven efficacy in humans, it has also been successfully tested in rats due to its action as a Type 5 phosphodiesterase (PDE5) inhibitor (Ballard et al. 1998; Corbin et al. 2002; Behr‐Roussel et al. 2005). PDE5 is responsible for the hydrolysis of cyclic guanosine monophosphate (cGMP), a key intracellular mediator of nitric oxide (NO)–induced smooth muscle relaxation. By inhibiting PDE5, sildenafil increases and prolongs cGMP signalling in the corpus cavernosum, thereby promoting cavernosal smooth muscle relaxation and increasing penile blood flow, which facilitates erection (Andersson 2001).

Despite the high incidence of erectile dysfunction in stallions, to the authors' knowledge there are no reports in the literature of pharmacological treatment specifically targeting erectile dysfunction in this species. Thus, the objective of the present study is to report the use of sildenafil citrate in two cases of aged stallions with erectile dysfunction.

## 2 Materials and Methods

This study was approved by the Ethics Committee on the Use of Production Animals of the Federal University of Viçosa (CEUAP—UFV), under protocol 87/2026.

### 2.1 Case Histories

Two aged stallions, 18 and 25 years old of the Quarter Horse and Mangalarga Marchador breeds, respectively, were referred to the Animal Reproduction Service of the FMVZ‐UNESP Veterinary Hospital (Botucatu, SP, Brazil) with a history of difficulty achieving erection and ejaculation.

Stallion 1, an 18‐year‐old Quarter Horse, had a previously satisfactory reproductive history with good performance during his fertile years. However, in the last 3 years he began to require progressively more intense sexual stimuli to ejaculate, such as longer exposure to a mare and the need to use different mares. As the condition progressed, he started having difficulty ejaculating, requiring two to four mounts to complete the act. In the past year the problem worsened, with incomplete erections and multiple ejaculatory failures, progressing in the last 8 months to an inability to maintain a sufficient erection for semen collection.

**FIGURE 1:**
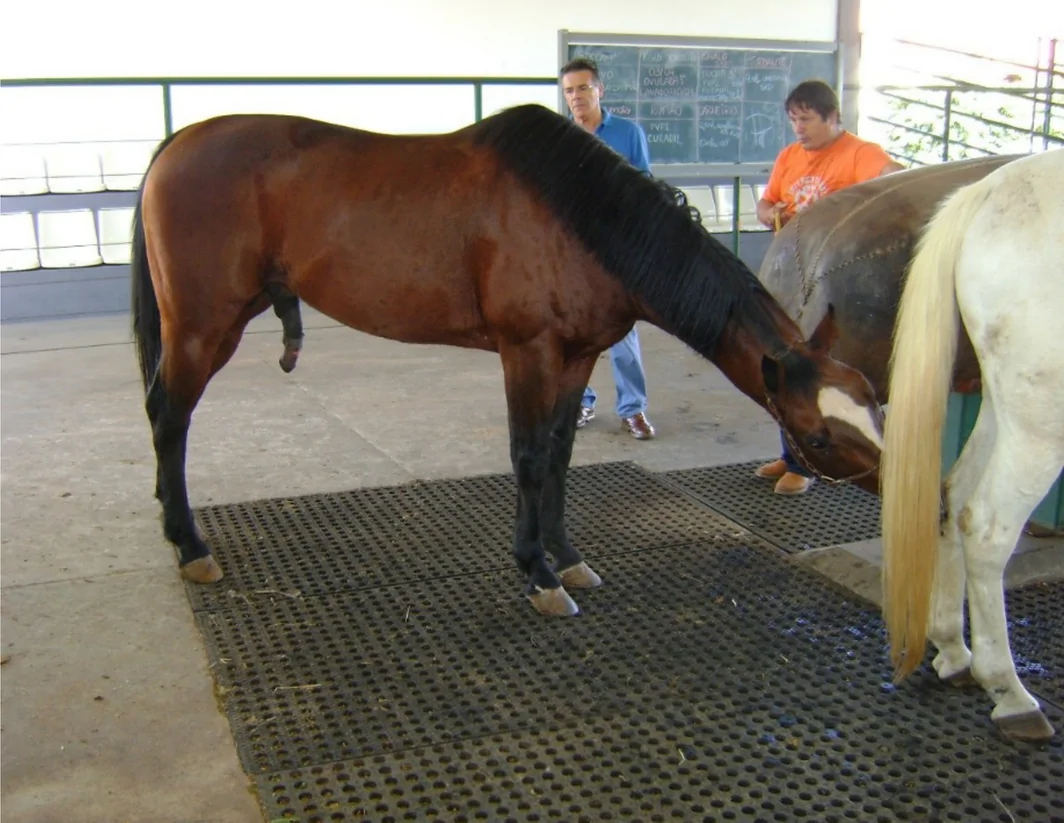
Quarter Horse stallion showing normal libido but incomplete erection when exposed to a mare in estrus, before sildenafil citrate treatment.

A thorough physical examination of Stallion 1 revealed no musculoskeletal abnormalities, lameness, ataxia, locomotor disorders, neurological deficits, or lumbar pain on palpation. A complete reproductive examination confirmed the integrity of the genital tract, with no abnormalities to explain the problem. The prepuce and penis showed no visible lesions, and both testes and epididymides were symmetrical, freely movable within the scrotum, with consistency and position normal for the species. After exposure to an estrous mare to evaluate erectile response, an incomplete erection was observed (Figure 1) despite the stallion showing interest in the female. Even with the use of an artificial vagina, the degree of penile rigidity remained insufficient to allow ejaculation. Ultrasonographic examination revealed normal testicular echotexture, with homogeneous echogenicity and a pampiniform plexus vascular pattern within physiological limits, as described by Pozor (2007). The accessory sex glands also showed sonographic images consistent with normality, according to Pozor and McDonnell (2002).

Stallion 2, a 25‐year‐old Mangalarga Marchador, had a previously satisfactory reproductive history prior to a surgical procedure. However, after undergoing colic surgery 2 years ago, he began to have difficulty fully retracting the penis into the prepuce. The physical exam revealed no musculoskeletal abnormalities, signs of lameness, ataxia, locomotor deficits, neurological deficits or back pain. A complete andrological exam confirmed the integrity of the reproductive system: the prepuce and penis were intact, and both testes and epididymides were symmetrical and freely movable in the scrotum with normal texture and position. However, both testes were flaccid on palpation, suggesting a degenerative process. Ultrasonography indicated normal testicular echogenicity and preserved pampiniform plexus blood flow, as described by Pozor (2007), and the accessory sex glands appeared normal, as reported by Pozor and McDonnell (2002).

### 2.2 Semen Collection Attempts

The stallions were individually exposed to an estrous mare, and semen collection attempts were performed using a Botucatu‐model artificial vagina (Botupharma Ltd., Botucatu, SP, Brazil), varying temperature (45°C–60°C), pressure and use of lubricant. Manual stimulation at the base and glans of the penis with warm compresses was also applied, in addition to attempting semen collection with the stallion mounting a mare, a phantom dummy and on the ground, as described by McDonnell and Love (1990).

Even after all these strategies, and despite retained libido, both stallions would extend the penis without achieving full erection or ejaculation. For the purposes of this case report, erection was considered incomplete when the penis failed to achieve sufficient rigidity to permit intromission or to be maintained within the artificial vagina despite penile extension and the presence of normal sexual interest toward an estrous mare. Based on these findings, the stallions were diagnosed with erectile dysfunction.

### 2.3 Treatment

After the diagnosis was confirmed, both stallions were treated with sildenafil citrate (Viagra, Pfizer) at a dose of 0.2 mg/kg orally. Tablets were ground and dissolved in 5 mL of distilled water, and the solution was administered via a 10 mL syringe (with the tip removed) directly in the stallion's stall, 30 min before sexual stimulation.

The sildenafil citrate dose used in the present case report (0.2 mg/kg, orally) was selected as a conservative, safety‐oriented approach, considering the absence of previous dose–response studies in stallions with erectile dysfunction. The selected dose was also informed by a translational approach based on the established clinical use of sildenafil in men, in whom the recommended oral dose for erectile dysfunction generally ranges from 25 to 100 mg, with 25 mg commonly considered a reduced initial dose in elderly patients or in individuals who may be more susceptible to drug‐related effects. Although direct interspecies dose extrapolation has clear limitations, this information was used as a clinical reference to support the selection of a low initial dose in these aged stallions. Thus, the dose adopted in the present cases was intended to provide a pharmacological stimulus while minimizing the potential risk of adverse effects.

Thirty minutes after treatment, three semen collection attempts were performed on consecutive days using the same methodology described above.

The stallions were clinically monitored for potential adverse effects following sildenafil administration. Twenty‐five minutes after administration, the animals were evaluated for changes in general behaviour and for alterations in heart and respiratory rates. Also, after completion of the semen collection attempts, the stallions were allowed to rest and were reassessed 40 min later for general behaviour, heart rate and respiratory rate to monitor for potential delayed adverse effects associated with sildenafil administration.

### 2.4 Semen Evaluation

After collection, the semen was filtered through gauze to remove the gel fraction and impurities, and diluted in a skim milk‐based extender pre‐warmed to 37°C (BotuSemen, Botupharma Ltd.) to adjust the concentration to 50 × 10^6 spermatozoa/mL. Sperm motility parameters were evaluated by a CASA system (HTM‐IVOS 12, Hamilton‐Thorne Research, USA), as described by Cavalero et al. (2019), analysing total motility (TM, %), progressive motility (PM, %) and the percentage of rapid spermatozoa (RAP, %).

An aliquot of raw semen was used to determine sperm concentration, using a Neubauer haemocytometer chamber (Optik Labor, Lancing, UK) under phase‐contrast microscopy (Jenamed 2, Carl Zeiss, Munich, Germany) at 200× magnification.

Plasma membrane integrity (MI) was assessed by epifluorescence microscopy (Leica DMLB, Wetzlar, Germany), using a combination of the probes carboxyfluorescein diacetate (CFDA) and propidium iodide (PI), as described by Harrison and Vickers (1990). A total of 100 cells were evaluated; spermatozoa showing completely green fluorescence were considered to have an intact plasma membrane, whereas those showing red or combined green/red fluorescence were considered to have a damaged plasma membrane.

## 3 Results

Stallion 1 (18‐year‐old Quarter Horse) achieved full erection 30 min after treatment with sildenafil citrate (SC) and ejaculated successfully in all semen collection attempts (100%, 3/3). In each attempt, ejaculation occurred after three to five mounts on the dummy, and the semen parameters were within normal ranges for the species according to the Brazilian College of Animal Reproduction (CBRA 2013) guidelines (Table 1).

**TABLE 1:** Mean values of seminal characteristics from semen collections in an 18‐year‐old Quarter Horse stallion (Stallion 1) and a 25‐year‐old Mangalarga Marchador stallion (Stallion 2) with erectile dysfunction, treated with sildenafil citrate.

Parameter	Stallion 1	Stallion 2
Volume (mL)	53.3	14
Sperm concentration (×10^6/mL)	76.7	35
Total sperm count (×10^9)	3.4	0.5
Mounts (per ejaculation)	4	5
Total motility (%)	73.3	21
Progressive motility (%)	35.0	13
Rapid spermatozoa (%)	66.3	14
Plasma membrane integrity (%)	50.3	26
Attempts resulting in ejaculation (*n* = 3)	3	1

*Note:* 0 = no ejaculation in three attempts; 1 = ejaculated once in three attempts; 2 = ejaculated in two of three attempts; 3 = ejaculated in all three attempts.

Stallion 2 (25‐year‐old Mangalarga Marchador) showed a visible improvement in erection 30 min after SC treatment, although it did not reach a complete erection. Despite this partial response, the stallion ejaculated in one out of the three attempts (33.3%), after five mounts on the dummy in each trial. The quality of the semen obtained exhibited oligozoospermia and parameters below the standard established by the CBRA (2013) (Table 1).

No behavioural changes or adverse clinical signs were observed during the evaluations performed 25 min after sildenafil administration and 40 min after completion of the semen collection attempts. At both time points, heart and respiratory rates remained within the physiological ranges for the species.

## 4 Discussion

In stallions with erectile dysfunction, manual stimulation with warm compresses can be used as a technique to obtain an ejaculate (McDonnell and Love 1990). However, in the stallions of the present study no improvement in erection or sexual arousal was observed with manual stimulation combined with warm compresses at the base of the penis before starting drug treatment. This result corroborates a previous report describing ejaculatory difficulty in the presence of incomplete erection, leading to successive unsuccessful attempts (McDonnell 1999). These observations support the clinical relevance of adequate penile erection for successful semen collection and ejaculation in stallions, although erection itself is not considered essential for the ejaculatory reflex (McDonnell 2000).

In the present study, an improvement in erectile capacity was evident in both stallions thirty minutes after SC administration. In Stallion 1, a marked improvement in penile erection was observed when he was exposed to a mare in estrus. The increase in erection, noted both on visual examination and tactile evaluation of the penis, was temporally associated with SC administration and suggests a potential improvement in erectile function. This response is consistent with the known pharmacological action of sildenafil through PDE5 inhibition and enhancement of NO/cGMP‐mediated cavernosal smooth muscle relaxation, as demonstrated in humans and rodents (Ballard et al. 1998; Corbin et al. 2002). However, to our knowledge, PDE5 expression and the specific effects of sildenafil on the NO/cGMP pathway have not been directly characterized in equine corpus cavernosum. Therefore, although the clinical response observed in these stallions is compatible with this mechanism, its occurrence in equine penile tissue cannot be confirmed from the present cases. NO induces relaxation of smooth muscle in both the corpus cavernosum and the penile vascular endothelium, allowing complete engorgement of the penis with blood, as shown in figure 1 (Ballard et al. 1998; Corbin et al. 2002).

**FIGURE 2:**
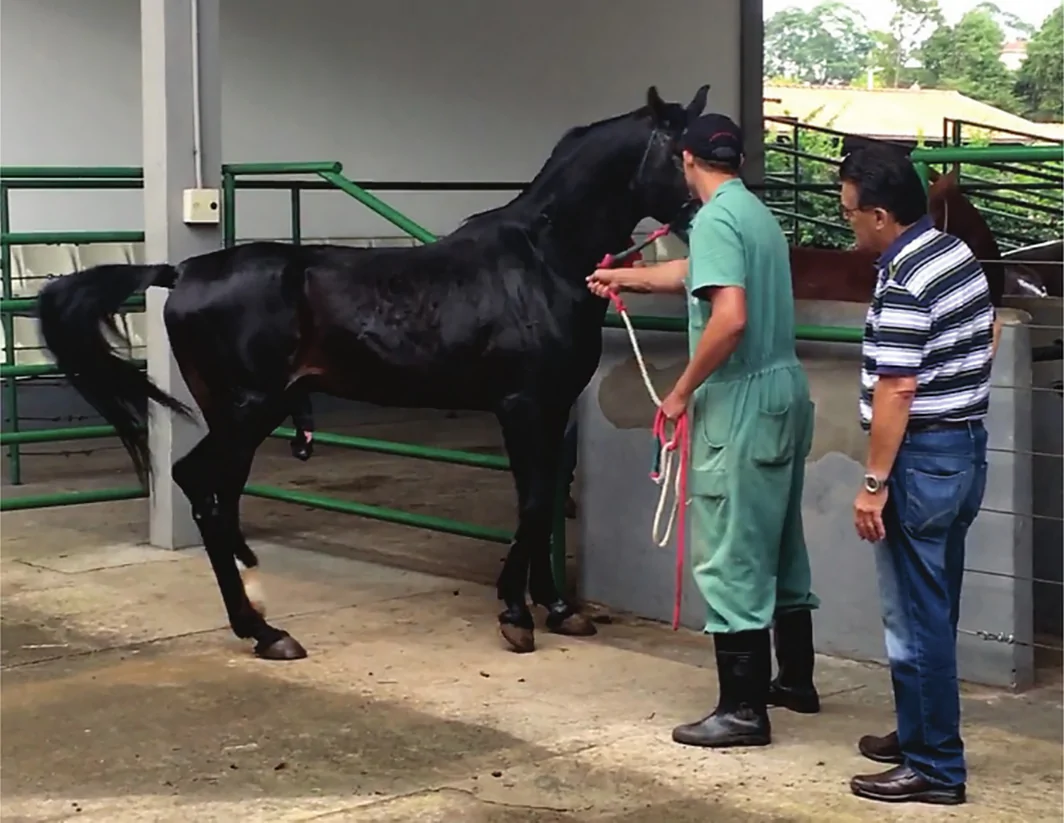
Mangalarga Marchador stallion showing normal libido but incomplete erection when exposed to a mare in estrus before sildenafil citrate treatment.

Stallion 2 showed a partial improvement of the erectile response after treatment; however, a full penile erection was not achieved (Figure 2). This incomplete erection possibly resulted in insufficient somatosensory stimulation of the glans penis, which may have contributed to the fact that the animal achieved ejaculation in only one of the three attempts. The insufficient erectile response could be related to the relatively low dosage of sildenafil citrate used in the experimental protocol. The variation in response between the two stallions may also indicate individual differences in pharmacodynamic sensitivity to sildenafil.

In humans, sildenafil is primarily used in elderly patients, as erectile dysfunction in older men has been linked to decreased NO and cGMP production, thereby hindering the initiation of the erection process (Ballard et al. 1998; Corbin et al. 2002). Age‐associated changes in erectile physiology could potentially have contributed to the dysfunction observed in the two aged stallions included in this report; however, this remains speculative because the mechanisms underlying age‐related erectile dysfunction have not been specifically characterized in stallions. A decline in reproductive performance in stallions typically occurs beyond 10 years of age (Dowsett and Knott 1996). Studies in rats have found similar results, with improved erection and a reduced time to ejaculation after SC administration (Behr‐Roussel et al. 2005).

Aside from the improvement in penile erection observed after treatment, the ejaculates obtained from Stallion 1 had semen parameters within the expected ranges for the species (Table 1) in accordance with equine species standards (Sieme 2009). However, because semen collection was unsuccessful before sildenafil administration, pre‐treatment seminal parameters were unavailable for either stallion. Therefore, it is not possible to determine whether sildenafil had any positive or negative effect on semen characteristics, and the post‐treatment seminal values should be interpreted only as descriptive findings. Consequently, no inference regarding an effect of SC on accessory sex gland function or sperm quality can be made from these cases. Administration of PGF_2_α in stallions results in ejaculates with greater volume, likely due to a stronger stimulation of the seminal vesicles during copulation, and it can cause urospermia, sweating and colic, as previously reported after its use (McDonnell 1992).

In Stallion 2, the partial improvement in erection following sildenafil administration, associated with successful ejaculation in one of the three semen collection attempts, supports the interpretation that erectile dysfunction was one component of the reproductive impairment observed in this stallion. Sildenafil was administered specifically to address the erectile dysfunction and was not expected to improve the concurrent testicular condition. The low sperm concentration, reduced total sperm count and flaccid testicular consistency observed during the andrological examination are more consistent with age‐related testicular degeneration than with erectile dysfunction itself. Therefore, these findings suggest the coexistence of two distinct conditions: erectile dysfunction, which limited successful semen collection, and testicular degeneration, which was likely responsible for the reduced semen quality. Nevertheless, because no pre‐treatment ejaculate could be obtained, the contribution of sildenafil to the seminal characteristics observed after treatment cannot be determined. Thus, the poor semen quality should not be interpreted as either an adverse effect or a lack of effect of sildenafil.

Given the absence of pharmacokinetic and pharmacodynamic studies establishing an optimal dose of sildenafil in horses, a conservative dose of 0.2 mg/kg was selected to minimize the potential risk of adverse effects. This approach was also informed by the clinical use of relatively low initial sildenafil doses in elderly men or in patients who may be more susceptible to the effects of the drug, while recognizing the limitations of direct interspecies dose extrapolation. Accordingly, the dose used in the present cases should be considered empirical, since equine‐specific information regarding oral bioavailability, plasma half‐life, effective tissue concentrations and dose–response relationships is currently unavailable. Therefore, the different erectile responses observed between the two stallions cannot be used to infer that the dose was suboptimal for Stallion 2.

No clinically apparent adverse effects were observed in either stallion following sildenafil administration, and heart and respiratory rates remained within physiological ranges during the evaluations performed 25 min after administration and 40 min after completion of the semen collection attempts. Nevertheless, these observations represent only short‐term clinical monitoring. Blood pressure was not evaluated, and the limited number of animals and short observation period preclude conclusions regarding the cardiovascular or overall safety of sildenafil in stallions.

Finally, the present report should be interpreted as preliminary clinical evidence rather than demonstration of therapeutic efficacy. The absence of untreated controls, the inclusion of only two stallions, the lack of pre‐treatment semen samples, and the absence of equine‐specific pharmacokinetic and pharmacodynamic data limit causal interpretation of the observed responses. Nevertheless, the temporal association between sildenafil administration and improvement in penile erection, particularly the complete erectile response and successful ejaculation observed repeatedly in Stallion 1, supports further investigation of PDE5 inhibition as a potential pharmacological approach for erectile dysfunction in stallions. Controlled studies including larger numbers of animals, objective assessment of erectile response, pharmacokinetic characterization and dose–response evaluation are warranted.

## 5 Conclusion

To the best of the authors' knowledge, this is the first report describing the administration of sildenafil citrate for erectile dysfunction in stallions. In these two cases, sildenafil citrate was associated with improved penile erection and enabled ejaculation, although the response varied between individuals. Further controlled studies are required to evaluate its efficacy and safety and to establish appropriate therapeutic doses for the treatment of erectile dysfunction in stallions.

## Author Contributions

**Thaís Mendes Sanches Cavalero:** conceptualization, investigation, methodology, data curation, writing – original draft, writing – review and editing. **Frederico Ozanam Papa:** supervision, project administration, resources, formal analysis, writing – review and editing. **Gabriel Augusto Monteiro:** conceptualization, investigation, methodology, data curation, writing – original draft, writing – review and editing. **Patrícia de Mello Papa:** investigation, formal analysis. **Felipe Pires Hartwig:** investigation, formal analysis. **Camila de Paula Freitas‐Dell'Aqua:** data curation, formal analysis. **Marco Antônio Alvarenga:** investigation, methodology, formal analysis. **Marcela Souza e Freitas:** investigation, formal analysis. **Yame Fabres Robaina Sancler‐Silva:** conceptualization, investigation, methodology, data curation, writing – original draft, writing – review and editing.

## Conflicts of Interest

The authors declare no conflicts of interest.

## Data Availability

The data that support the findings of this study are available from the corresponding author upon reasonable request.

## References

[rda70318-bib-0001] Andersson, K. E. 2001. “Neurophysiology/Pharmacology of Erection.” International Journal of Impotence Research 13, no. 3: 8–13. 10.1038/sj.ijir.3900718 11477487

[rda70318-bib-0002] Ballard, S. A. , C. J. Gingell , K. I. M. Tang , L. A. Turner , M. E. Price , and A. M. Naylor . 1998. “Effects of Sildenafil on the Relaxation of Human Corpus Cavernosum Tissue In Vitro and on the Activities of Cyclic Nucleotide Phosphodiesterase Isozymes.” Journal of Urology 159, no. 6: 2164–2171. 9598563 10.1016/S0022-5347(01)63299-3

[rda70318-bib-0003] Behr‐Roussel, D. , D. Gorny , K. Mevel , et al. 2005. “Chronic Sildenafil Improves Erectile Function and Endothelium‐Dependent Cavernosal Relaxations in Rats: Lack of Tachyphylaxis.” European Urology 47, no. 1: 87–91. 15582254 10.1016/j.eururo.2004.09.005

[rda70318-bib-0004] Cavalero, T. M. S. , F. O. Papa , R. A. Schmith , et al. 2019. “Protocols Using Detomidine and Oxytocin Induce ex Copula Ejaculation in Stallions.” Theriogenology 140: 93–98. 31454723 10.1016/j.theriogenology.2019.08.024

[rda70318-bib-0019] Cavalero, T. M. S. , L. G. T. M. Segabinazzi , V. F. C. Scheeren , L. E. F. Canuto , M. L. M. Gobato , and F. F. Papa . 2020. “Alternative Protocol Using Imipramine, Detomidine, and Oxytocin for Semen Collection in Stallion with Ejaculatory Dysfunction.” Journal of Equine Veterinary Science 93: 103205. 32972673 10.1016/j.jevs.2020.103205

[rda70318-bib-0005] CBRA—Colégio Brasileiro de Reprodução Animal . 2013. Manual para exame andrológico e avaliação de sêmen animal. 3ª ed. CBRA.

[rda70318-bib-0006] Corbin, J. D. , S. H. Francis , and D. J. Webb . 2002. “Phosphodiesterase Type 5 as a Pharmacologic Target in Erectile Dysfunction.” Urology 60, no. 2: 4–11. 10.1016/s0090-4295(02)01686-2 12414329

[rda70318-bib-0007] Dowsett, K. F. , and L. M. Knott . 1996. “The Influence of Age and Breed on Stallion Semen.” Theriogenology 46, no. 3: 397–412. 16727908 10.1016/0093-691x(96)00162-8

[rda70318-bib-0020] Garza, J. , S. Del Angel , M. F. Gallelli , and M. Miragaya 2025. “Pharmacologically Induced Ex Copula Ejaculation in Stallions: Assessment of New Combined Protocols.” Journal of Equine Veterinary Science 145: 105288.

[rda70318-bib-0008] Harrison, R. A. P. , and S. E. Vickers . 1990. “Use of Fluorescent Probes to Assess Membrane Integrity in Mammalian Spermatozoa.” Journal of Reproduction and Fertility 88: 343–352. 1690300 10.1530/jrf.0.0880343

[rda70318-bib-0009] Klug, E. , C. P. Bartmann , and H. Gehlen . 1999. “Diagnosis and Therapy of Copulatory Disorders of the Stallion.” Pferdeheilkunde 15, no. 6: 494–502.

[rda70318-bib-0010] McDonnell, S. M. 1992. “Ejaculation: Physiology and Dysfunction.” Veterinary Clinics of North America. Equine Practice 8, no. 1: 57–70. 1576554 10.1016/s0749-0739(17)30466-2

[rda70318-bib-0011] McDonnell, S. M. 1999. “Libido, Erection, and Ejaculatory Dysfunction in Stallions.” Compendium on Continuing Education for the Practicing Veterinarian 21, no. 8: 862–871.

[rda70318-bib-0012] McDonnell, S. M. 2000. “Reproductive Behavior of Stallions and Mares: Comparison of Free‐Running and Domestic In‐Hand Breeding.” Animal Reproduction Science 60: 211–219. 10844196 10.1016/s0378-4320(00)00136-6

[rda70318-bib-0018] McDonnell, S. M. 2001. “Oral Imipramine and Intravenous Xylazine for Pharmacologically‐Induced Ex Copula Ejaculation in Stallions.” Animal Reproduction Science 68, no. 3–4: 153–159. 11744260 10.1016/s0378-4320(01)00152-x

[rda70318-bib-0013] McDonnell, S. M. , and C. C. Love . 1990. “Manual Stimulation Collection of Semen From Stallions: Training Time, Sexual Behavior and Semen.” Theriogenology 33, no. 6: 1201–1210.

[rda70318-bib-0017] Monteiro, G. A. , and I. M. Petelinkar . 2025. “Ejaculatory Disorders and Advanced Semen Collection Techniques in Stallions.” Revista Brasileira de Reprodução Animal 49, no. 2: 688–695.

[rda70318-bib-0014] Pozor, M. A. 2007. “Evaluation of Testicular Vasculature in Stallions.” Clinical Techniques in Equine Practice 6, no. 4: 271–277.

[rda70318-bib-0015] Pozor, M. A. , and S. M. McDonnell . 2002. “Ultrasonographic Measurements of Accessory Sex Glands, Ampullae, and Urethra of Normal Stallions of Various Size Types.” Theriogenology 58, no. 7: 1425–1433. 12387354 10.1016/s0093-691x(02)01034-8

[rda70318-bib-0016] Sieme, H. 2009. “Semen Evaluation.” In Equine Breeding Management and Artificial Insemination, edited by J. C. Samper , 2nd ed. St. ed., 57–74. Saunders (Elsevier).

